# Correlation Between Volume and Pressure of Intracranial Space With Craniectomy Surface Area and Brain Herniation: A Phantom-Based Study

**DOI:** 10.1089/neur.2024.0006

**Published:** 2024-03-27

**Authors:** Sudip Kumar Sengupta, Rohit Aggarwal, Manish Kumar Singh

**Affiliations:** ^1^ Department of Neurosurgery, Command Hospital Southern Command (Pune), India.; ^2^ Department of Radiology, Command Hospital Southern Command (Pune), India.; ^3^ Department of Anaesthesia, Command Hospital Southern Command (Pune), India.

**Keywords:** decompressive craniectomy, intracranial pressure, phantom and CT scan-based study, volume and pressure changes

## Abstract

There are proponents of decompressive craniectomy (DC) and its various modifications who claim reasonable clinical outcomes for each of them. Clinical outcome in cases of traumatic brain injury, managed conservatively or aided by different surgical techniques, depends on multiple factors, which vary widely among patients and have complex interplay, making it difficult to compare one case with another in absolute terms. This forms the basis of the perceived necessity to have a standard model to study, compare, and strategize in this field. We designed a phantom-based model and present the findings of the study aimed at establishing a correlation of the volume of intracranial space and changes in intracranial pressure (ICP) with surface area of the craniectomy defect created during DC and brain herniation volume. A roughly hemispherical radio-opaque container was scanned on a 128-slice computed tomography scanner. Craniectomies of different sizes and shapes were marked on the walls of the phantom. Two spherical sacs of stretchable materials were subsequently placed inside the phantom, fixed to three-way connectors, filled with water, and connected with transducers. The terminals of the transducer cables were coupled with the display monitor through a signal amplifier and processor module. Parts of the wall of the phantom were removed to let portions of the sac herniate through the defect, simulating a DC. Volume measurements using AW volume share 7^®^ software were done. Resection of a 12.7 × 11.5 cm part of the wall resulted in a 10-cm-diameter defect in the wall. Volume differential of 35 mL created a midline shift of 5 mm to the side with lesser volume. When measuring pressure in two stretchable sacs contained inside the phantom, there always remained a pressure differential ranging from 1 to 2 mm Hg in different recordings, even with sacs on both sides containing an equal volume of fluids. Creating a circular wall defect of 10 cm in diameter with an intracavitary pressure of 35 mm Hg on the ipsilateral sac and 33 mm on the contralateral sac recorded with intact walls, resulted in a true volume expansion of 48.411 cm^3^. The herniation resulted in a reduction of pressure in both sacs, with the pressure recorded as 25 mm in the ipsilateral sac and 24 mm in the contralateral sac. The findings closely matched those of the other model-based studies. Refinement of the materials used is likely to provide a valid platform to study cranial volume, ICP, craniectomy size, and brain prolapse volume in real time. The model will help in pre-operatively choosing the most appropriate technique between a classical DC, a hinge craniotomy, and an expansive cranioplasty technique in cases of refractory raised ICP.

## Introduction

Decompressive craniectomy (DC) has been the most commonly performed surgical procedure for refractory raised intracranial pressure (ICP) of varied etiology.^1^ Various modifications of DC have been proposed with a desire to make it a single-stage procedure.^2–6^ Clinical outcome in cases of traumatic brain injury (TBI), managed conservatively or aided by different surgical techniques, depends on multiple factors, which vary widely among patients and have complex interplay, making it difficult to compare one case with another in absolute terms.^7^ Given that the intracranial volume changes evidenced on computed tomography (CT) scans and ICP are most easily and objectively measurable variables that help prognosticate and plan surgery in cases of TBI, much work has been done in these fields.^8–11^ It is, however, well documented that clinical variables far outweigh the CT-based predictors in isolation in prognosticating outcome.^12^ It is also known that ICP monitoring does not offer anything superior to monitoring other clinical parameters.^13^ To add to the variability, the cranial cavity differs widely in volume between persons, making it impractical to extrapolate findings of one case to another.^14^ (The volume and shape of intracranial cavities vary widely among humans. A minimum of 1398 mL to a maximum of 1830 mL has been reported by Bradley and colleagues.)

We designed a phantom-based model with an intention to provide a standardized environment where different objective parameters could be assessed for their inter-relationship. We present the findings of the study on this phantom-based model to establish a correlation of the volume of intracranial space and changes in ICP with the surface area of the craniectomy defect created during DC and brain herniation volume. We have also performed a literature search to discover and compare other models developed with a similar objective.

## Methods

### The phantom

**FIG. 1.**
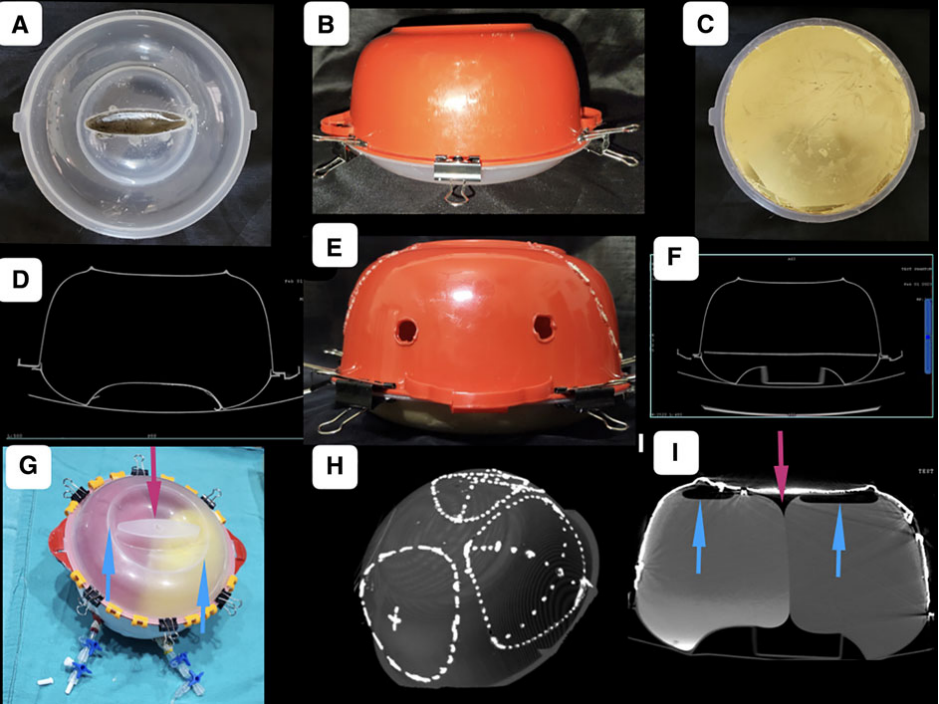
The phantom. A roughly hemispherical radio-opaque container (we used a plastic container) was used as a phantom for the study. (**A**) Lid of the container of 400 mL in volume. (**B**) Container of 1900 mL in volume with a lid applied on it. (**C**) Volume of the lid obliterated by pouring plaster of Paris paste/molten wax (**D**) and (**F**) cross-section of the phantom as seen on CT scan with the volume adjusted at 1900 and 1500 mL, respectively. (**E,H**) Craniectomies of different sizes and shapes were marked on the walls of the phantom by drilling holes. The holes were plugged by radiocontrast containing putty for better visualization on the CT scan. Two holes made on the side walls to allow connecting the stretchable sacs with three-way stoppers. (**G,I**) Phantom with two fluid-filled stretchable sacs, as seen by the naked eye and on the CT scan. Blue and red arrows depict entrapped air inside and outside the sacs, respectively. CT, computed tomography.

A roughly hemispherical radio-opaque container (we used a plastic container) was used as a phantom for the study (Fig. 1). Its volume could be adjusted between 1500 and 1900 mL by adjusting the base. In our study, we poured a measured volume of plaster of Paris paste/molten wax into the container to elevate the base and reduce its working volume as desired. Walls of the phantom were presumed to be representing the cranium and its volume: the cranial cavity. The volume was measured physically by filling the cavity up with a measured quantity of water.

### Dimensional and volumetric measurements

**FIG. 2.**
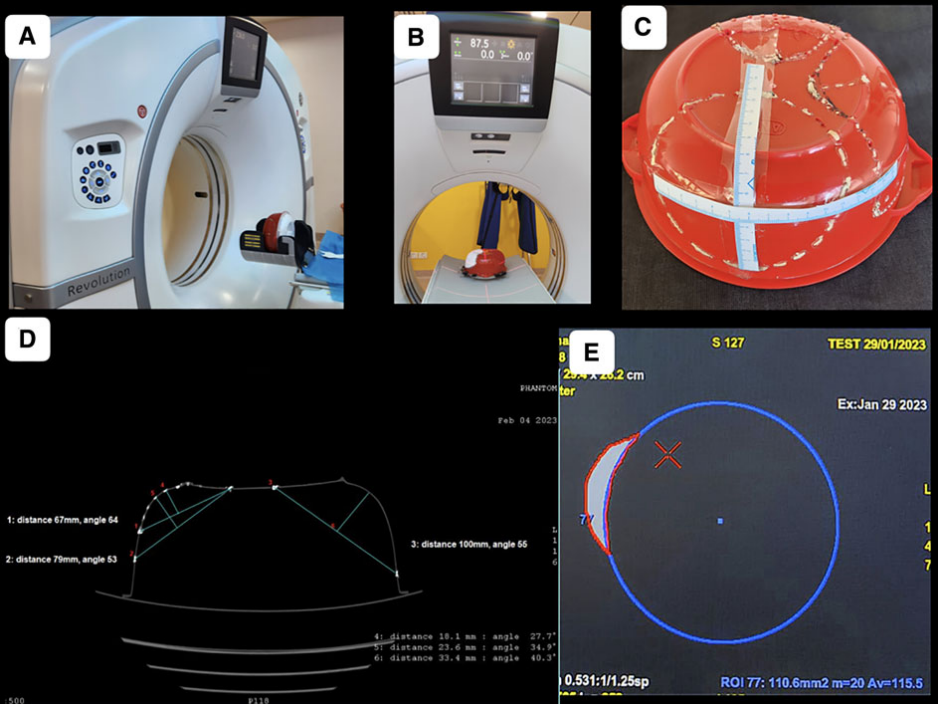
Dimensional and volumetric measurements. Phantoms were scanned on a 128-slice CT scanner. (**A,B**) The phantom was placed on the CT scan gantry in vertical and horizontal positions and scanned. (**C**) Various measurement on markings representing craniectomies of different sizes and shapes drilled on the walls of the phantom were done by measuring tapes. (**D**) Radio-opaque material plugged holes, drilled on the walls visible as hyperdense dots and the plastic wall of the container as thin hyperdense lines on 1.25-mm thin reconstructions of CT scan images. (**E**) To calculate distance, circumference, surface area, and volume on CT scan images, AW volume share 7^®^ software was used. CT, computed tomography.

Phantoms were scanned on a 128-slice CT scanner (Revolution EVO; GE Healthcare, Milwaukee, WI) in helical mode under the head CT protocol using 110 kVp and variable effective mAs (Fig. 2). Advantage workstation (AW)^®^ version 4.7 was used for post-processing of data. Then, 1.25-mm thin reconstructions were generated in both the soft tissue window and bone window. The phantom was placed on the CT scan gantry in vertical and horizontal positions (Fig. 2A,B). Craniectomies of different sizes and shapes were marked on the walls of the phantom by drilling holes. Various measurements were done on the phantom by measuring tapes (Fig. 2C). The holes were plugged by radiocontrast containing putty for better visualization on the CT scan (Fig. 2D). Two spherical sacs of stretchable materials were subsequently placed inside the phantom through two holes (Fig. 2D). The sacs were fixed to three-way connectors and filled with water until they expanded to occupy nearly the entire space available, barring entrapped air (Fig. 2E). Studies were repeated with a single sac and with different working volumes (Fig. 2F). To calculate all the measurements and volume, AW volume share 7^®^ software was used.

### Intracavitary pressure measurements

**FIG. 3.**
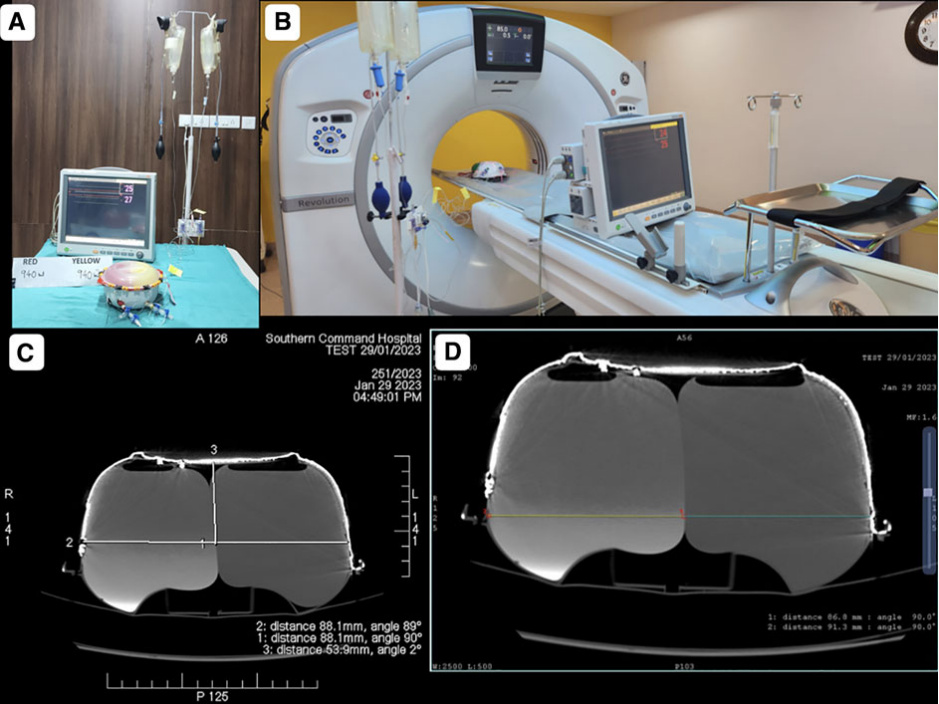
Intracavitary pressure measurements. (**A**) Assembled circuit for pressure measurement showing a three-way Stopcock placed at the outlet of each balloon connected with fluid-filled (normal saline) pressure-monitoring tubings while the third port of the Stopcock was used for altering the fluid volume of the balloons. The other end of the pressure-monitoring tubings was connected with transducers. Each transducer was also connected with a pressure flushing system and a cable. The terminals of the cables were coupled with the display monitor through a signal amplifier and processor module. The monitor was set to display pressures both in waveform and numerical values independently for each balloon. (**B**) Phantom with a pressure-monitoring assembly in the CT scan machine. (**C**) Two stretchable sacs filled up with an equal volume (915 mL each) of fluid without any MLS. (**D**) MLS (5-mm) on differential volumes with 950 mL on the sac on the right side and 915 mL on the left sac. CT, computed tomography; MLS, midline shift.

One 3-way Stopcock was placed at the outlet of each balloon and connected with fluid-filled (normal saline) pressure-monitoring tubings (Fig. 3). The third port of the Stopcock was used for altering the fluid volume of the balloons. The other end of the pressure-monitoring tubings was connected with transducers. Each transducer was also connected with a pressure flushing system and a cable. The terminals of the cables were coupled with the display monitor through a signal amplifier and processor module. The monitor was set to display pressures both in waveform and numerical values independently for each balloon.

### Computed tomography scan imaging-based volume measurement

**FIG. 4.**
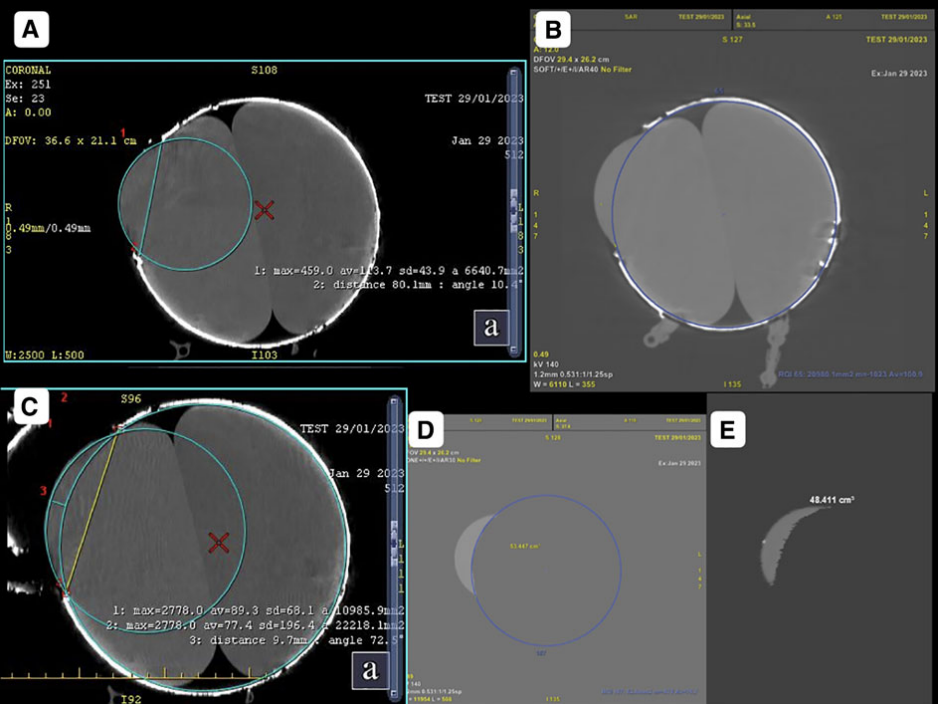
Calculation of herniated balloon volume using the planimetric method. (**A**) Circle drawn depicting the circumference of the herniated component of the balloon after decompression. (**B**) Line depicting an imaginary line of the non-herniated balloon (circle drawn along the circumference of the phantom, representing the cranial cavity in the axial section). (**C**) Perpendicular line depicting the maximum distance of herniation between an imaginary line of the non-herniated balloon and herniated balloon circumference line. (**D,E**) Volume estimation of the herniated component of the balloon using the planimetric method.

The volume estimation of the herniated part of the balloon was done using the planimetric method (Fig. 4). First, the circumference of the inner aspect of the container depicting the skull was drawn. This line at the level of the craniectomy depicted an imaginary line of the unherniated balloon. A second circle with a circumference line matching the herniated component was then drawn. The area between the imaginary non-herniated balloon and herniated balloon was sequentially marked across all slices showing the DC. This marked volume was then calculated by the system and noted.

Different parameters were measured on the images acquired on three different models twice by three authors independently and once with all of them sitting together. The median of seven readings was taken as correct for each parameter, with the only exception being the volume measurements by the planimetric method, which was done only on one phantom and only by the radiologist.

## Results

### Cost of the model

The model for *in vitro* study cost Rs 400.00 (4.84 USD). All the components are available in the local market.

### Validity of the model

All the tests were conducted on three different sets of models and results evaluated by the three authors separately. On one occasion, all three authors sat together and did the measurements in consultation with each other. Results were closely reproduced each time with a maximum difference of 1.2 mm when measuring linear distance and 36.0 mL while measuring the volume of the cavity of the phantom (depicting the intracranial cavity).

### Shape of the cavity in the phantom representing the intracranial cavity

**FIG. 5.**
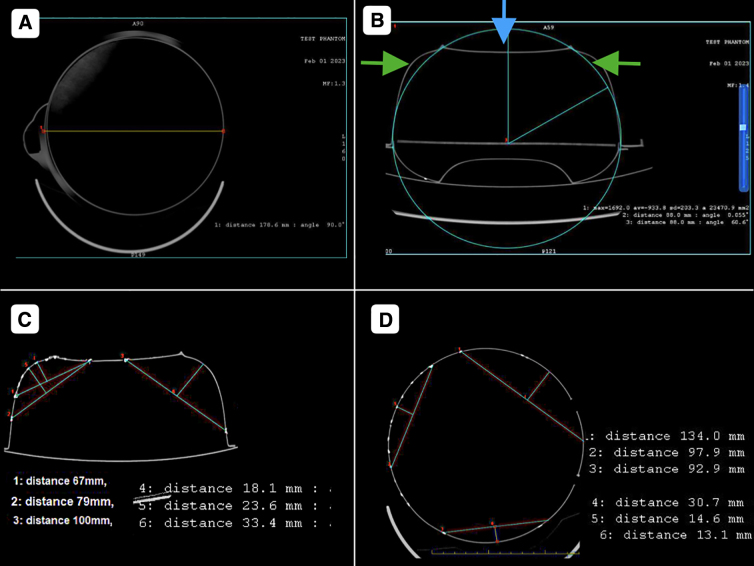
Measurements on CT scan images. (**A,B**) A 1500-mL volume of the phantom has an 8.93-cm radius of the base, and an 8.80-cm-radius hemisphere can represent it. Any depression (green arrows) at a given point has been compensated by corresponding bulge(s) at other points (blue arrow). (**C,D**) Measurements in the axial and coronal plane of the distance of the inner table from the level of craniectomy and the corresponding diameter of the defect. CT, computed tomography.

A 1500-mL volume of the phantom had an 8.93-cm radius of the base, and an 8.80-cm-radius hemisphere could represent it. Any depression at a given point had been compensated by corresponding bulge(s) at other points (Fig. 5A,B).

The maximum distance from the line joining the craniectomy margins to the outer margin of unexpanded dura was found to be greater when measured in the axial section (which represents the human skull in the coronal plane) than that measured in the coronal plane (representing the human skull in the axial plane) in the phantom (Fig. 5C,D).

Resection of a 12.7 × 11.5 cm part of the wall resulted in a 10-cm-diameter defect in the wall (Fig. 7).

**FIG. 6.**
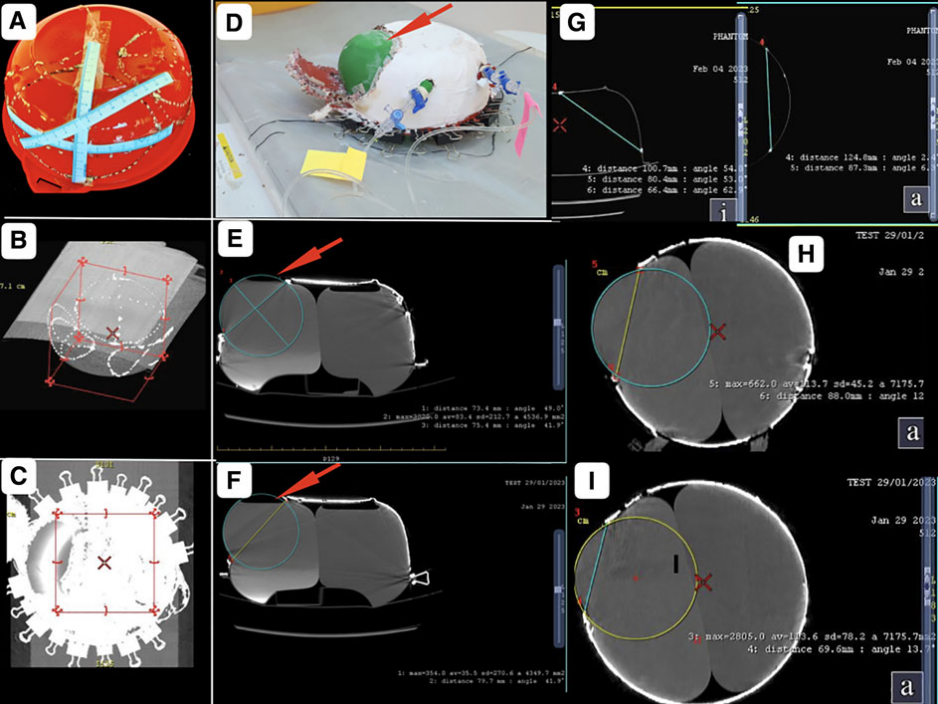
Wall defect and fluid-filled sac herniation. (**A,B**) Markings on the phantom representing an oval craniectomy with the 11.6 × 14 cm bone flap and its volumetric reconstructions of CT scan iamges. (**C**) The 3D volumetric reconstruction of the CT scan image showing an out-pouching of underlying fluid-filled sac herniating through the oval defect created on removing the flap. (**D**) Phantom placed horizontally in the CT gantry with the 14.0 × 11.6 cm oval flap of its wall removed, resulting in out-pouching of the green stretchable fluid-filled sac through the defect. (**E,F**) Axial sections of the CT scan image showing the out-pouchings through a circular wall defect to be part of an imaginary circle of the surface area 4536.9 mm^2^ with the center of the circle moving laterally as one approaches the center of the defect. (**H,I**) Coronal reconstruction of images showing out-pouchings through a circular wall defect to be forming part of the imaginary circles of an area of 7175.7 cm^2^. (**G**) CT scan measurements showing a 10.07 × 12.46 cm defect created by removing an 11.6 × 14.0 cm overlying flap. Red arrows indicate the trapped air bubble inside the sac. 3D, three-dimensional; CT, computed tomography.

**FIG. 7.**
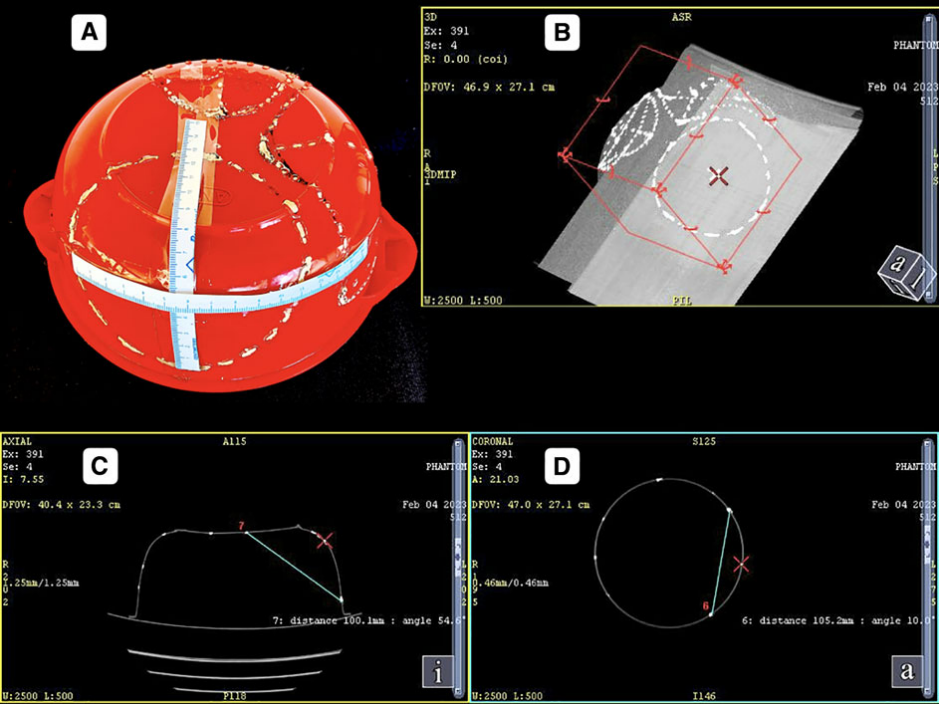
A 10-cm-diameter wall defect. (**A**) The 12.7 × 11.5 cm flap marked over the wall of the phantom. (**B**) The 3D reconstruction of CT scan image with the pointer on the 12.7 × 11.5 cm flap. **(C,D)** Axial craniectomy defect diameter of 10 cm as measured on two orthogonal planes for the 12.7 × 11.5 cm flap. 3D, three-dimensional; CT, computed tomography.

### Properties of the segment of sac projecting out of a defect in the wall representing brain herniation through craniotomy defect after expansive cranioplasty

In every orthogonal section, the portion of a sac projecting out from a containing cavity through a circular defect was found to resemble a section of an imaginary circle. The diameter of the circle remained constant for any particular circular craniotomy defect. The imaginary circle appeared to shift centrifugally as the image shifted from the poles toward the equator (Fig. 6E,F).

In the case of an oval wall defect, the projecting portion of the sac was not hemispherical. However, in each orthogonal section, it still appeared to resemble a section of an imaginary circle. The diameter of the circle, though, kept changing in accordance with the size of the wall defect recorded in that section, unlike a sac projecting out of a circular defect.

### Volume differential of 35 mL created a midline shift of 5 mm to the side with lesser volume

When measuring pressure in two stretchable sacs contained inside the phantom, there always remained a pressure differential ranging from 1 to 2 mm Hg in different recordings, when sacs on both sides contained an equal volume of fluids (Fig. 3D). Pressure differential remained in the same range even when a volume differential of 35 mL was created between them or decompression was achieved. The pressure recording was always greater on one of the sides throughout the study, and it did not matter which side was containing more fluid.

Creating a circular wall defect of 10 cm in diameter with an intracavitary pressure of 35 mm Hg on the ipsilateral sac and 33 mm on the contralateral sac, recorded with intact walls, resulted in a true volume expansion of 48.411 cm^3^ (Fig. 4E). The herniation resulted in a reduction of pressure in both sacs, with the pressure recorded as 25 mm in the ipsilateral sac and 24 mm in the contralateral sac.

## Discussion

DC has a time-proven role in reducing midline shift (MLS) and improving patient outcome in terms of both morbidity and mortality in cases of refractory raised ICP.^1^ Various modifications of DC have been proposed with an aim to reduce its complications, improve outcome, or make it a single-stage procedure.^2–6^ There are proponents of each of these diverse, sometimes apparently conflicting modifications, with claims of matching, if not superior, clinical outcomes over the traditional DC.^6^

There are some inherent issues with the intracranial cavity with regard to its volume, pressure, and content shifts that make comparison between any two cases difficult. First, the volume and shape of intracranial cavities vary widely among humans. A minimum of 1398 to a maximum of 1830 mL has been reported by Bradley and colleagues.^14^ Second, intracranial pressure measurements in the immediate pre-operative period, during different stages of DC and post-operative follow-up, are not routinely recorded using a single universally used technique.^15^ What is worse, DC itself is known to contribute various complications influencing the outcome.^16^ Third, the craniotomies designed are of varied shapes and, in fact, are hardly ever circular.^1^ This variation of shape and size makes it difficult to measure craniectomy surface areas and the volume of brain parenchyma herniated through it. So, this topic often remains untouched in many research works. Fourth, there are many pre-existing clinical conditions, patient variables, injury patterns, pre-injury relation of brain parenchyma volume to that of the intracranial cavity, and also the quality of nursing care, which are difficult to be factored in during the assessment of clinical outcome of DC and its variants.^17^ The above four and perhaps some more elements missed here make the comparison of different clinical trials or case series unrealistic.

This forms the basis of the perceived necessity to have a standard model to study, compare, and strategize in this field. Multiple efforts have been made by different authors to this effect.^18–20^

In the present study, we have been able to devise a three-dimensional (3D) model of a simple design and low cost (<5 USD) that can be improved upon in terms of material used, shape, and size. The hemispheric brain of 1500 cc in volume, represented by a half circle of 8.945 cm in diameter in the two-dimensional (2D) mathematical model, could be well represented by a 3D hemisphere of 8.80 cm (mean 8.90 cm) in the present study.

The portion of sac projecting out of a circular defect was found to be a segment of an imaginary sphere (Fig. 4A,B). For an elliptical wall defect, the portion of sac projecting out did not resemble a sphere. However, in each CT scan section, it did appear to be a segment of a circle, the size of which kept increasing with the size of the defect in that section (Fig. 6E,F,H,I). For a craniectomy defect with 10 cm in diameter, the lateral shift of the outer margin of the sac (h_2_) was recorded as 9.7 mm (Fig. 4C). This finding was similar to the study by Kwon and colleagues.^21^

The size of bone flap was larger than the craniectomy defect it created attributed to curvature of the skull, an observation similar to that made by Tanrikulu and colleagues.^22^ Further, we observed in our study that the discrepancy was greater for a flap that crossed convexity on the phantom representing the superior temporal line and increased more and more as it extended medially. Variance between the flap size and wall defect size kept increasing with the increase in flap size.

We found that the pressures measured in two stretchable fluid-filled sacs contained in the phantom were not identical when the containing walls were intact. Pressures measured among the pair of sacs were unequal in all the readings, with different positions of the phantom and different pressures at which measurements were made. The difference persisted even after creating a defect in the wall of the phantom allowing one of the sacs to prolapse. The difference was greater when intracavity pressure was increased by injecting fluids on one side. The difference was reduced once the wall defect presenting a DC was created. This is in consonance with the other reported studies on bilateral ICP measurement.^23^

The maximum distance between the inner table (h_1_) and plane of craniectomy in the present 3D model-based study (Fig. 4C) closely resembled that in the 2D mathematical model.^20^ In the present study, the h_1_, for a given craniectomy size, was greater for a bone flap that crossed the convexity on the phantom representing the superior temporal line and increased more and more as it extended medially. The h_1_ was also found to be increasing with the increase in craniectomy size. This measurement was substantially greater when measured in coronal sections than in the axial section for any wall defect size.

### Study limitations

Contents of the intracranial cavity have different density and elastic properties. Using a water-filled sac made of a stretchable material to represent intracranial contents in the present model is a serious drawback, especially when calculating brain prolapse volume.

We conducted the studies with the phantom cavity set at different volumes. The parameters reported upon in our study did not change in different working volumes. However, human skulls are of varying shapes, and changing shapes will have a varied effect on parameters in the clinical setting.

## Conclusion

This 3D hemispherical phantom-based study provides a workable arrangement of objectively measuring the surface area, volume, and pressure correlations of the intracranial cavity in real time. The stretchability of the sacs used was not standardized, and, of course, an oversimplified representation of the intracranial cavity contents having varying viscoelastic properties is always debatable. These drawbacks notwithstanding, the model did provide a first-of-its-kind opportunity to understand a few basics on the subject. The findings closely matched those of other model-based studies. Refinement of the materials used is likely to make it more reliable and useful. This model will help in pre-operatively choosing the most appropriate technique between a classical DC and one of its variants, in cases of refractory raised ICP.

## Transparency, Rigor, and Reproducibility Summary

The study was not registered because it did not involve any living creature or data pertaining to any human being, nor could it have caused any adverse event. It was a model-based study. There were no human or non-human participants in the study. Hence, consent/incomplete study/inadequate follow-up/withdrawal of consent/adverse effects/death never happened. Outcomes were recorded, as they happened, on the images acquired on three different models twice by three authors independently and once with all of them sitting together. The median of seven readings was taken as correct for each parameter, with the only exception being the volume measurements by the planimetric method, which was done only on one phantom and only by the radiologist. Results noted by one author were not communicated to the other when each of them were carrying out their part of the study. Data were acquired between September 16, 2022 and August 15, 2023 between 5:00 pm and 1:00 am. There were no tissue samples involved. Phantoms were scanned on a 128-slice CT scanner (Revolution EVO; GE Healthcare, Milwaukee, WI) in helical mode under the head CT protocol using 110 kVp and variable effective mAs. Advantage workstation^®^ version 4.7 was used for post-processing of data. Then, 1.25-mm thin reconstructions were generated in both the soft tissue window and bone window. The phantom was placed on the CT scan gantry in vertical and horizontal positions. Craniectomies of different sizes and shapes were marked on the walls of the phantom by drilling holes. Various measurements were done on the phantom by measuring tapes. All components of the phantom are widely available in local markets. The data reported in this study can be used in future researches without the requirement for specific additional informed consent.

## Abbreviations Used

2D: two-dimensional
3D: three-dimensional
CT: computed tomography
DC: decompressive craniectomy
ICP: intracranial pressure
MLS: midline shift
TBI: traumatic brain injury
